# Virtual reality simulation training in stroke thrombectomy centers with limited patient volume—Simulator performance and patient outcome

**DOI:** 10.1177/15910199231198275

**Published:** 2023-09-06

**Authors:** Olav Søvik, Arnstein Tveiten, Halvor Øygarden, Pål Johan Stokkeland, Hanne Brit Hetland, Magnus Sundgot Schneider, Knut Olav Sandve, Marianne Altmann, Dan Levi Hykkerud, Johanna Ospel, Mayank Goyal, Hege Langli Ersdal, Martin Wilhelm Kurz, Per Kristian Hyldmo

**Affiliations:** 1 Department of Research, Sørlandet Hospital, Kristiansand, Norway; 2 621781 Faculty of Health Sciences, University of Stavanger, Stavanger, Norway; 3 Department of Neurology, Sørlandet Hospital, Kristiansand, Norway; 4 Faculty of Health and Sport Sciences, 4343 University of Agder, Kristiansand, Norway; 5 Department of Radiology, Sørlandet Hospital, Kristiansand, Norway; 6 Department of Research, Section of Biostatistics, 60496 Stavanger University Hospital, Stavanger, Norway; 7 Department of Radiology, 60496 Stavanger University Hospital, Stavanger, Norway; 8 423433 Department of Neurology, Akershus University Hospital, Lørenskog, Norway; 9 Department of Radiology, Akershus University Hospital, Lørenskog, Norway; 10 Department of Radiology, 30262 Basel University Hospital, Basel, Switzerland; 11 Department of Clinical Neurosciences, 2129 University of Calgary, Calgary, Alberta, Canada; 12 Diagnostic Imaging, 2129 University of Calgary, Calgary, Alberta, Canada; 13 Department of Neurology, Neuroscience Research Group, 60496 Stavanger University Hospital, Stavanger, Norway; 14 Department of Clinical Science, 1658 University of Bergen, Norway

**Keywords:** Virtual reality, simulation training, endovascular thrombectomy, ischemic stroke, reperfusion therapy

## Abstract

**Background:**

Virtual reality simulation training may improve the technical skills of interventional radiologists when establishing endovascular thrombectomy at limited-volume stroke centers. The aim of this study was to investigate whether the technical thrombectomy performance of interventional radiologists improved after a defined virtual reality simulator training period. As part of the quality surveillance of clinical practice, we also assessed patient outcomes and thrombectomy quality indicators at the participating centers.

**Methods:**

Interventional radiologists and radiology residents from three thrombectomy-capable stroke centers participated in a five months thrombectomy skill-training curriculum on a virtual reality simulator. The simulator automatically registered procedure time, the number of predefined steps that were correctly executed, handling errors, contrast volume, fluoroscopy time, and radiation dose exposure. The design was a before-after study. Two simulated thrombectomy cases were used as pretest and posttest cases, while seven other cases were used for training. Utilizing the Norwegian Stroke Register, we investigated clinical results in thrombectomy during the study period.

**Results:**

Nineteen interventional radiologists and radiology residents participated in the study. The improvement between pretest and posttest cases was statistically significant for all outcome measures in both simulated cases, except for the contrast volume used in one case. Clinical patient outcomes in all three centers were well within the recommendations from multi-society consensus guidelines.

**Conclusion:**

Performance on the virtual reality simulator improved after training. Virtual reality simulation may improve the learning curve for interventional radiologists in limited-volume thrombectomy centers. No correlation alleged, the clinical data indicates that the centers studied performed thrombectomy in accordance with guideline-recommended standards.

## Introduction

Stroke is a leading cause of morbidity and mortality worldwide, and large vessel occlusion (LVO) strokes have a particularly high risk of poor outcomes.^1^ Endovascular thrombectomy (EVT) is the treatment of choice for LVO stroke, with a number needed to treat as low as 2.6 for improved clinical outcome.^2^ However, urgent treatment is critical for good outcomes, and delivering EVT to all eligible patients in a timely manner is a major challenge worldwide.^3^

EVT centers with high patient volumes may achieve better clinical outcomes for their EVT patients.^4^ However, in sparsely populated countries with long transfer distances like Norway, a careful trade-off between centralization and accessibility of acute stroke care must be made. To increase access to timely thrombectomy, Norwegian authorities have decided that some hospitals that are not comprehensive stroke centers shall become new EVT centers, sharing the underlying idea of the entity “Thrombectomy Capable Stroke Centers” in the United States.^5^ At these Norwegian centers, general interventional radiologists perform EVT.

To alleviate the issue of limited patient volume, these Norwegian thrombectomy-capable stroke centers collaborate in a network with a common quality improvement (QI) program. The QI program consists of continuous quality surveillance, simulation team training for the acute stroke team, and EVT task-training on a virtual reality (VR) simulator for the general interventional radiologists. A pilot study from one center in the QI program network showed that VR simulation task-training enhanced the capability to perform EVT in a VR simulation environment.^6^

Several studies have evaluated VR simulation task-training in diagnostic cerebral angiography,^7–9^ carotid artery stenting,^10–12^ and EVT.^13–16^ VR simulation has also been included as a complementary tool in training programs for interventional radiologists.^17,18^ However, the systematic use of VR simulators over time to improve EVT skills of general interventional radiologists has to our knowledge not been studied in a multi-center study.

The aim of the present multi-center study was to investigate if the VR simulator performance of general interventional radiologists improved after a defined VR simulation training period. In addition, we aimed to compare outcomes from clinical practice and other EVT quality metrics from the three centers with EVT quality benchmarks recommended by multi-society consensus guidelines.^19^

## Methods

The Regional Committee for Medical and Health Research approved the study. Informed written consent was obtained from the VR simulation participants, and data collected from the VR simulator software were anonymized. The study is reported against STROBE (Strengthening the reporting of observational studies in epidemiology) guidelines.^20^

### Context

All Norwegian emergency hospitals are organized in a common public health system. The participating stroke centers in this study were Stavanger University Hospital (SUS), Akershus University Hospital (AHUS) and Sørlandet Hospital Kristiansand (SSK). All three centers are located in the southern part of Norway. They have limited neurosurgical capabilities and general interventional radiologists perform EVT. SUS is serving a population of 369,000, SSK a population of 310,000, and AHUS a population of 1,020,000. SSK and AHUS started performing EVT in 2019, SUS in 2009. None of the centers has performed more than 50 EVTs per year, except for the year 2019, when 56 EVTs were performed at SUS.

### QI program description

The acute stroke teams in the QI program participate in regular simulation team-training followed by debriefing by qualified facilitators.^21^ Feedback from debriefings is used for optimizing local EVT protocols. For the general interventional radiologists, the QI program in addition consists of an educational program including the European Diploma in Ischemic Stroke Intervention (EDSI)^22^ or other programs which include a visiting trainee period at a comprehensive stroke center similar to the one required for EDSI. The program also includes systematic EVT training on a VR simulator, consisting of 5 months of training with a minimum of 30 cases performed. Dedicated stroke nurses and stroke neurologists prospectively register the clinical results of performed stroke treatments in the unique, statutory national quality register for stroke treatment, the Norwegian Stroke Register.^23^

### VR simulation study

General interventional radiologists and radiology residents volunteered to participate. The study consisted of simulated EVT on a VR task-training simulator. The design was a before-after study with a pretest, a simulator-training period, and a posttest.

We used a Mentice VIST® G5 simulator (Mentice AB, Gothenburg, Sweden), which is a validated metric-based VR simulator.^24,25^ Introduction and handling of catheters and endovascular devices in the simulator leads to tactile feedback and visual feedback on a monitor. The simulator software includes nine endovascular stroke cases with Middle Cerebral Artery occlusions in both hemispheres. The cases include aortic arch types 1, 2, and 3.^26^ The software automatically registers metric-based data parameters. These parameters include predefined consecutive procedural steps completed, and possible handling errors, that is, predefined deviations from optimal operator performance.^25^ A detailed description is provided in Supplemental Tables S1 and S2.

Outcome measures in this study were time from start to end of the procedure (s), the number of accomplished steps that were technically correctly executed, the number of handling errors, applied intra-arterial contrast volume (ml), fluoroscopy time and patient radiation dose exposure measured as kerma area product (mGy).^27^

**Figure 1.**
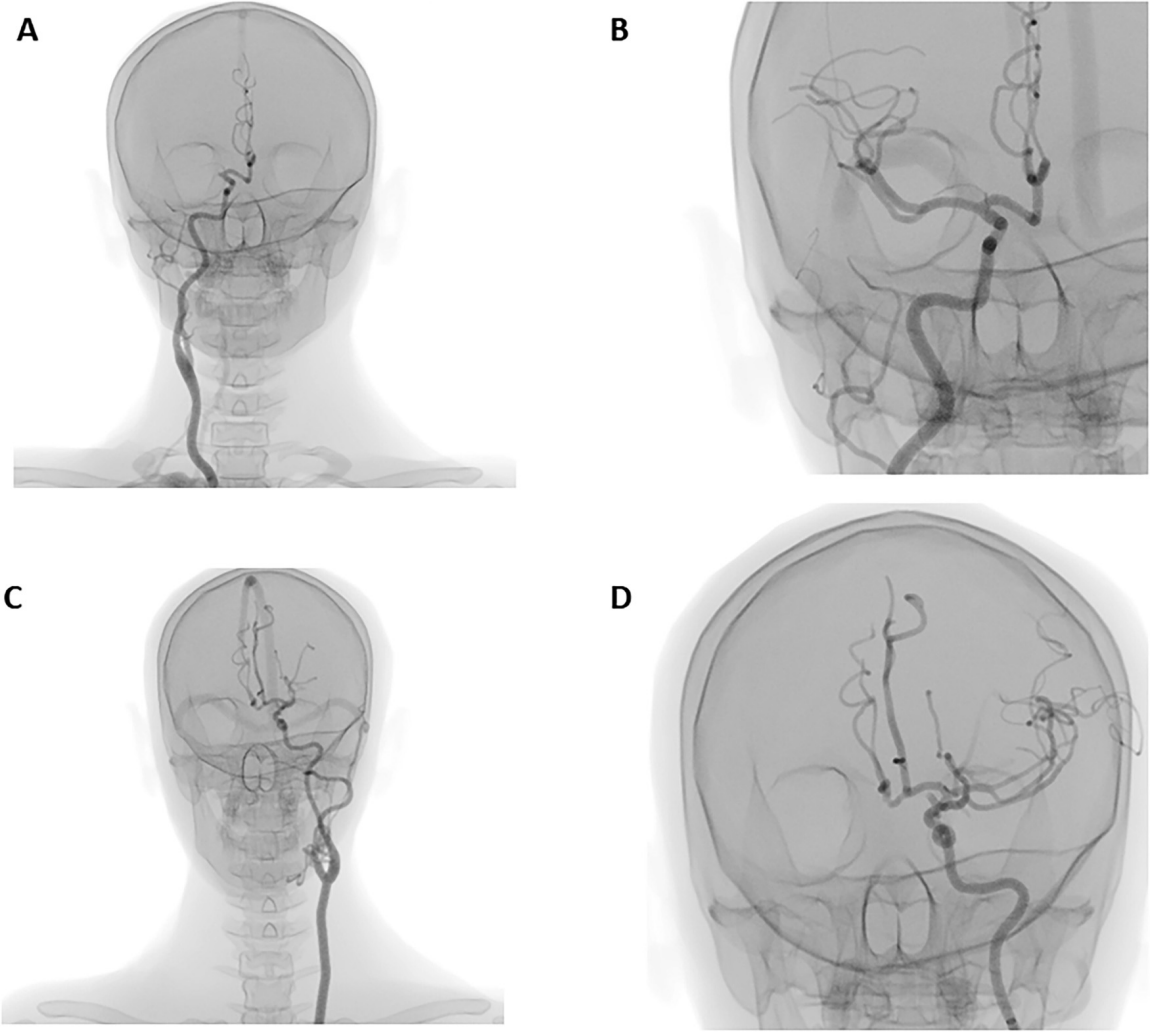
Examples of angiography images from the two test cases: (A) test case 1 before endovascular thrombectomy (EVT); (B) test case 1 after EVT; (C) test case 2 before EVT; (D) test case 2 after EVT.

Two of the nine endovascular stroke cases were selected for pretest and posttest use only (Figure 1). The first case was a patient with a type 1 aortic arch, while the second case had a more difficult type 3 aortic arch. Both had occlusion of the middle cerebral artery (M1). The remaining seven cases were available for training.

The study training period was from October 2019 to February 2020 at SUS, from December 2020 to April 2021 at SSK, and from March 2021 to August 2021 at AHUS. The participants performed the two pretest cases immediately prior to the five months training period. Each participant was instructed to perform a minimum of 30 simulated training cases during this period. The participants could choose freely from the seven training cases, and no upper limit of performed cases was defined. The VR simulator was placed in a dedicated room in each center, available to all participants at all times. The participants performed the two posttest cases immediately after the training period, which were the same two cases that were used in the pretest.

### Outcome from clinical practice

Dedicated stroke nurses and stroke neurologists continuously register the clinical results of EVT treatment at the three centers. The results are reported to the Norwegian Stroke Register.

As part of our QI programs quality surveillance of clinical practice, we assessed brain tissue reperfusion quality after EVT, hemorrhagic complications, and clinical outcomes. We compared these to multi-society consensus guidelines.^19^ The aim was early detection if any technical or clinical results at our centers should fall below international standards.

Data were collected from the Norwegian Stroke Registry from 2019 to 2021. Missing data were supplemented from patient records through a manual search. Brain tissue reperfusion after EVT was measured by the Modified Treatment in Cerebral Ischemia (mTICI) score.^28^ Hemorrhagic complications were measured by the frequency of symptomatic intracranial hemorrhage (sICH).^29^ Clinical outcomes were measured by Modified Rankin Scale (mRS) score^30^ 3 months after EVT.

### Statistical analysis

Statistical analyses were performed using SPSS Statistics version 26 (IBM Cooperation, Armonk, NY, USA). Numerical results were described with means and standard deviations. Pretest and posttest differences between variables were examined using paired samples *t*-test. In case of a negative correlation between pretest and posttest, independent samples *t*-tests were used.^31^ For the independent sample *t*-test, if the standard deviation in one sample was > 50% larger than in the other sample, Welch correction was applied. Levene's test was used to investigate if the posttest variance was lower than the pretest variance for the parameters measured. A significance level of 0.05 was used to determine statistical significance. As the participants were a convenience sample of volunteers, no formal power estimation was performed.

## Results

**Figure 2.**
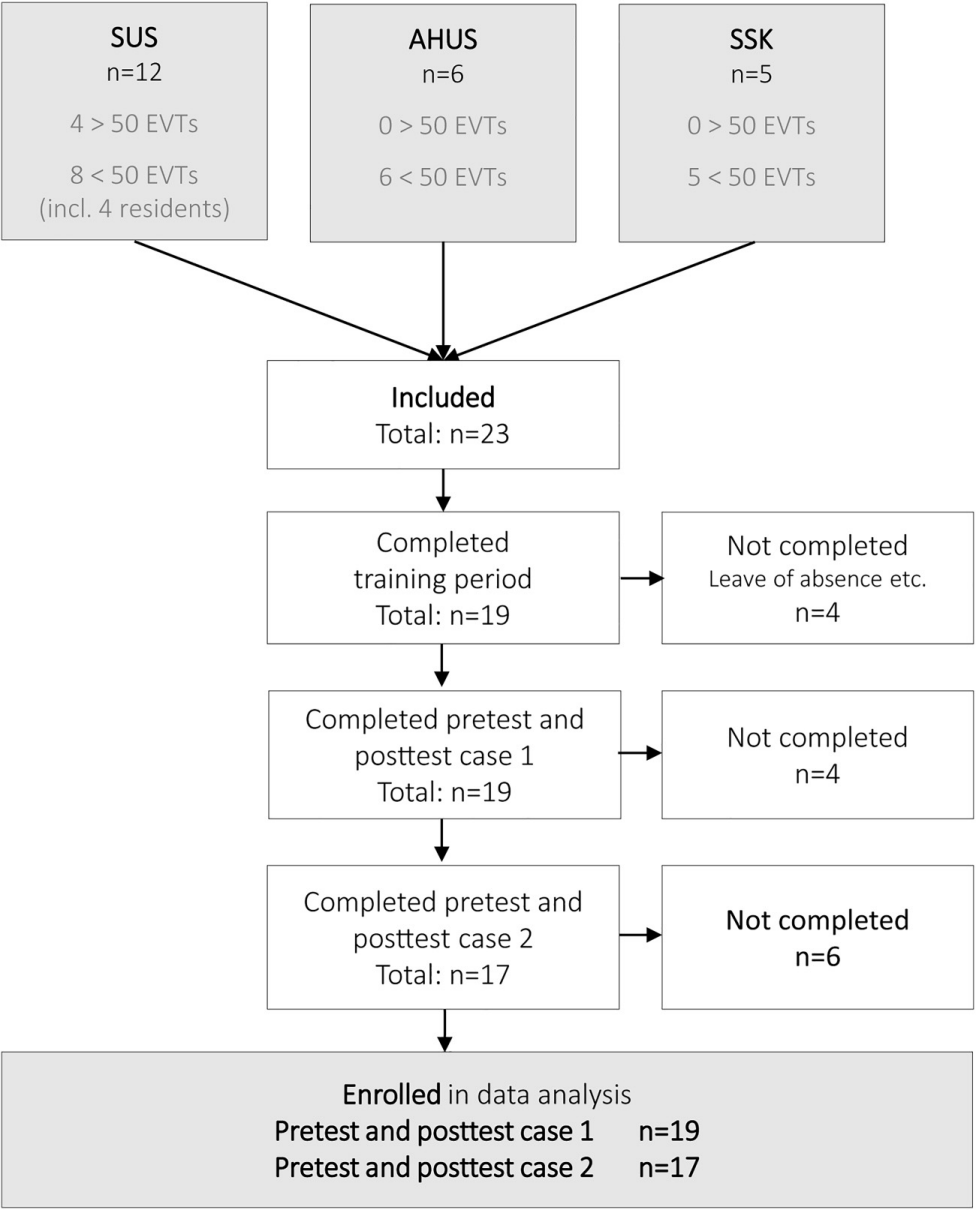
Participant's inclusion and completion of pretest, training, and posttest.

Twenty-three interventional radiologists and radiology residents were recruited, including all but one of the interventional radiologists at the three centers. Only four had previously performed more than 50 clinical EVTs, the remaining had performed 0–50 clinical EVTs. Four participants did not complete the training because of study leave or moving to another city. Two participants completed the pretest and posttest case 1, but not case 2 (Figure 2).

*n*: number; EVTs: number of clinical endovascular treatments performed before the study start.

The median number of performed training cases was 38, with a range from 12 to 91 cases. All but one performed at least 30 training cases. Two of the participants did the two posttest cases after less than five months of training; however, both had performed more than the target of 30 training cases before posttest cases.

### Pretest and posttest assessment

**Figure 3.**
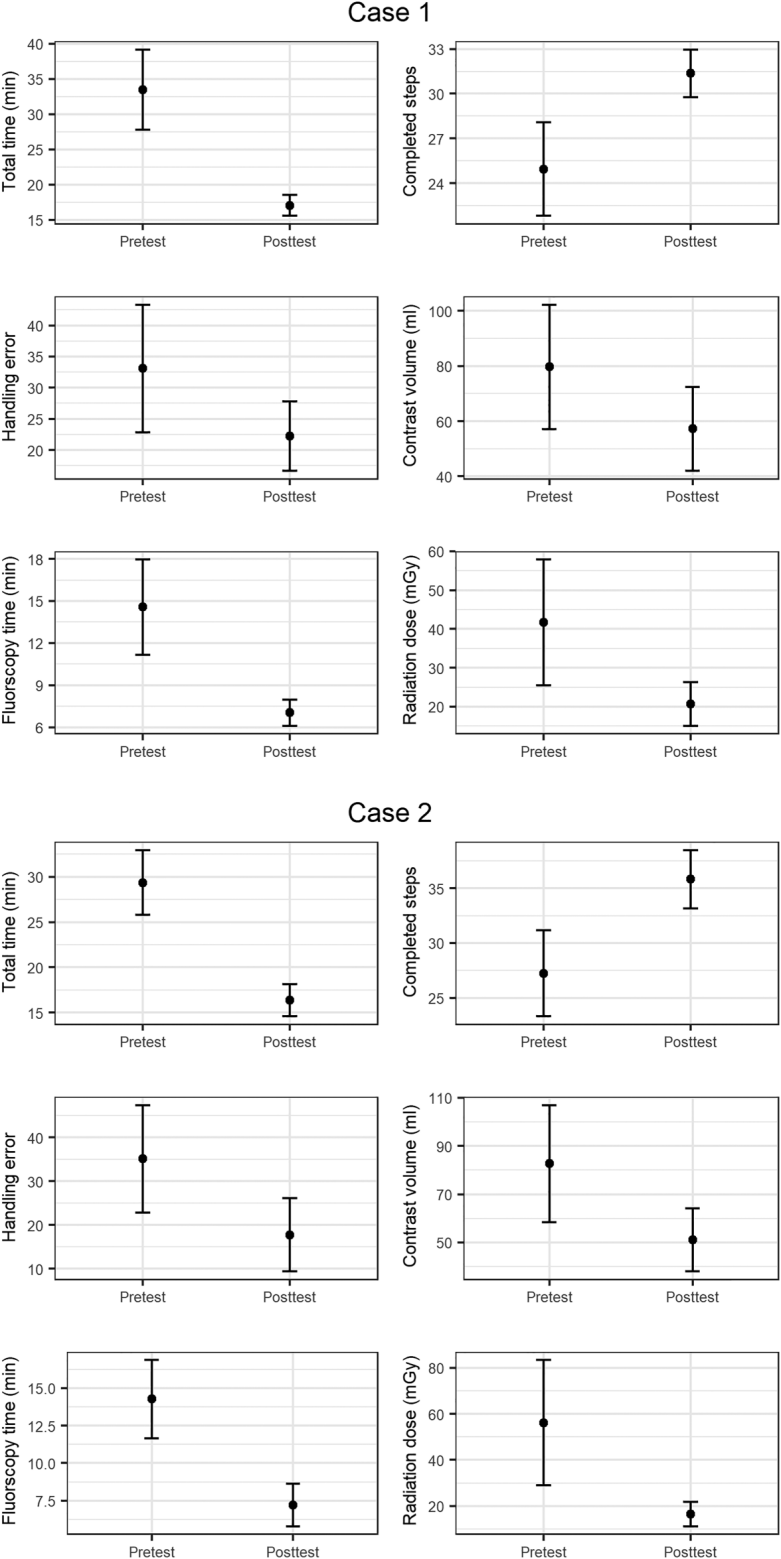
Comparison of the virtual reality (VR) simulator outcome measures in case 1 and case 2 pretest versus posttest.

The mean improvement between the pretest and posttest was statistically significant for all outcome measures in both cases, except for contrast volume used in case 1, where there was a non-significant trend towards improvement (Table 1, Figure 3, and Supplemental Table S3).

Data are presented as mean and 95% confidence interval.

**Table 1.** Comparison of the VR simulator parameters in pretest versus posttest for the test cases.

	Case 1 (*n* = 19)			Case 2 (*n* = 17)		
	Pretest (SD)	Posttest (SD)	*p*-value [CI]	Pretest (SD)	Posttest (SD)	*p*-value [CI]
Total time (min)	33.5 (11.9)	17.1 (3.1)	<0.001 [−21.4, −11.4]	29.4 (7.0)	16.4 (3.5)	<0.001^a^ [−16.9, −9.1]
Steps completed (*n*)	25.0 (6.5)	31.4 (3.3)	0.001 [3.0, 9.8]	27.2 (7.7)	35.8 (5.2)	0.001^b^ [4.0, 13.2]
Handling errors (*n*)	33.1 (21.3)	22.2 (11.6)	0.029 [−20.6, −1.2]	35.1 (23.9)	17.8 (16.3)	0.005 [−28.7, −6.0]
Contrast volume (ml)	79.7 (47.0)	57.2 (31.7)	0.072 [−47.2, 2.2]	82.7 (47.5)	51.1 (25.6)	0.024 [−58.3, −4.8]
Fluoroscopy time (min)	14.6 (7.1)	7.0 (1.9)	<0.001 [−10.9, −4.1]	14.3 (5.1)	7.2 (2.8)	<0.001 [−9.7, −4.4]
Radiation dose (mGy)	41.7 (33.8)	20.7 (11.7)	0.003 [−33.8, −8.1]	56.2 (53.2)	16.5 (10.4)	0.004 [−64.5, −14.9]

All values are given in mean (SD: standard deviation). *n*: numbers; CI: 95% confidence interval; ml and mGy are fictive values calculated from the simulator; VR: virtual reality.

^a^ Welch *t*-test.

^b^ Independent *t*-test.

The variance was significantly lower posttest than pretest for all parameters in case 1 except contrast volume, where there was a non-significant trend towards reduction. In case 2, there was a significant reduction in variance for total time, fluoroscopy time, and radiation dose, while there was a non-significant trend toward reduction for steps completed, handling errors, and contrast volume (Supplemental Table S4).

### Outcomes in clinical practice

The general interventional radiologists at the three centers, including most of the study participants, performed EVT on 191 patients during the study period from 2019 to 2021. The clinical reperfusion rates (mTICI ≥ 2b/3) were 85% or higher, proportions of patients achieving functional independence (mRS 0–2) were 44% or higher, and complication rates (sICH) were 5% or lower. The details for each center are presented in Table 2. Patients with pre-stroke mRS > 2, end-stage cancer, or more than one EVT performed during the same admission were excluded from this table, but are included in Supplemental Table S5.

**Table 2.** Clinical results from 2019 to 2021.

Number of patients	Brain reperfusion (mTICI ≥ 2b/3)	Independent at 3 months (mRS 0-2)	Hemorrhagic complications
SUS (*n* = 125)	86%	44%	4%
SSK (*n* = 27)	85%	48%	4%
AHUS (n = 39)	87%	49%	5%
Multisociety consensus guidelines^18^	> 70%	> 30%	< 10%

*n*: number of patients; mTICI: modified treatment in cerebral ischemia score; mRS: modified Rankin scale score; sICH: symptomatic intracranial hemorrhage.

## Discussion

In this study, at three thrombectomy-capable stroke centers, VR simulator training was associated with improved simulator performance in all measured technical skills. The improvement was statistically significant for all the assessed parameters in both test cases, except for the contrast volume used in one of the two cases. Furthermore, the variance was reduced in most measured parameters after training, which may indicate that VR simulator training was able to reduce “negative outliers.”^32^

The annual number of performed clinical EVTs at our hospitals is lower than recommended by some international professional societies^33,34^ and most of the general interventional radiologists in this study had limited clinical experience in performing EVT. The rationale for using VR simulators was thus to improve and accelerate learning for individual technical EVT skills,^21^ the ultimate goal being to improve patient outcomes. This is comparable to aviation pilots, who use VR simulators to improve and maintain their flying skills.^35^

VR simulation training offers operators the possibility to practice their skills in a safe environment and can effectively help to reduce complications in surgical and interventional specialties. Indeed, several studies have shown improved procedural and clinical outcomes of VR simulation for a variety of procedures, for example, elective carotid angiography,^36^ endovascular procedures,^37^ and laparoscopic surgery.^38^ In the neurointerventional field, simulation training for novice operators has been reported to be associated with significantly shorter fluoroscopy times, total procedure times, and reduced amount of contrast agent.^7,9,39^ Our results further support the usefulness of VR simulation for improving operators’ technical skills. However, although simulation is an integral part of the training curriculum in many surgical specialties, it is not yet widely used in interventional or neurointerventional radiology. To our knowledge, Norway is one of few countries in which routine simulation-based training is available for interventional radiology trainees.

Successful treatment of stroke patients requires much more than a skilled operator in the angiography lab. Coordinated and time-efficient teamwork among all members of the medical team, including the prehospital ambulance team, emergency department staff, stroke neurologists, nurses, and angiography suite team is important for a patient's outcome. Thus, the QI program at our hospitals also includes simulation team-training for the whole acute stroke team. At SUS, this training has been associated with faster initiation of treatment and improved patient outcomes. Median door-to-needle time in thrombolysis was significantly reduced from 27 to 13 min and remained consistent after 13 months.^40^ A combination of VR simulation training to improve technical skills and simulation team training to improve coordination and communication among all members of the medical team may therefore contribute effectively to improving workflow times, reperfusion quality, and most importantly, clinical patient outcomes.

Our QI program includes the recording of clinical data from EVT patients, and during the study period, clinical results from all three centers were in accordance with multi-society guideline recommendations.^19^ Our study was however not designed to assess a causal relationship between VR simulation, real-life EVT metrics, and patient outcomes.

Our study has limitations. First, we did not include a control group of physicians that did not undergo simulator training. Second, other factors than the simulator training, such as the learning effect of EVTs performed in real patients during the training period may have improved the participants’ skills.

However, at SSK and AHUS the simulation training took place during the early phase of clinical EVT implementation, and therefore, the overall number of EVTs in real patients during the training period was low (17 in SUS, four in SSK, and three in AHUS). Third, some of the enrolled participants did not complete the study because of study leave or moving to another city. Fourth, identical cases were chosen for the pre- and posttest cases to allow for direct comparability of parameters. Thus, there is a small chance that participants have memorized some of the case specifics, although the time between the pre-test and post-test was long and several VR simulator cases were performed in between.

In conclusion, VR simulation may help to improve the learning curve for general interventional radiologists when performing EVT in centers with a limited number of cases. In countries with sparse populations and long transfer distances such as Norway, minimizing time loss before EVT is often not compatible with the long transport times to a high-volume comprehensive stroke center. Simulation-based quality improvement programs such as the one presented in this study could contribute to solving this dilemma.

## Supplemental Material

sj-pdf-1-ine-10.1177_15910199231198275 - Supplemental material for Virtual reality simulation training in stroke thrombectomy centers with limited patient volume—Simulator performance and patient outcome Supplemental material, sj-pdf-1-ine-10.1177_15910199231198275 for Virtual reality simulation training in stroke thrombectomy centers with limited patient volume—Simulator performance and patient outcome by Olav Søvik, Arnstein Tveiten, Halvor øygarden, Pål Johan Stokkeland, Hanne Brit Hetland, Magnus Sundgot Schneider, Knut Olav Sandve, Marianne Altmann, Dan Levi Hykkerud, Johanna Ospel, Mayank Goyal, Hege Langli Ersda, Martin Wilhelm Kurz and Per Kristian Hyldmo in Interventional Neuroradiology
