# Supplementary material for: Virtual reality simulation training in stroke thrombectomy centers with limited patient volume—Simulator performance and patient outcome
Source: Interv Neuroradiol. 2023 Sep 6;32(1):85–92. doi: 10.1177/15910199231198275 (PMC12852635; doi:10.1177/15910199231198275)
Supplement: sj-pdf-1-ine-10.1177_15910199231198275 - Supplemental material for Virtual reality simulation training in stroke thrombectomy centers with limited patient volume—Simulator performance and patient outcome [file sj-pdf-1-ine-10.1177_15910199231198275.pdf]

Table S1. EVT procedure Case 1 deconstructed into consecutive steps and possible handling errors.

**** = Predefined possible handling errors***

I Femoral access to descending aorta

Advance wire in a smooth, continuous movement to mid thoracic aorta

- Advancing wire under fluoro, always keeping tip in view, until above celiac trunk and below left subclavian.

Advance BGC and selective catheter over the wire, under fluoroscopy, keeping tip of wire in view

- Advance BGC and 5F to mid thoracic aorta and proximal to flexible tip of wire

NOTE: In the simulator BGC must first be introduced to at least above celiac trunk before introducing the 5F

- * ***tool in side branch***
- * ***wire tip out of view***
- * ***distal tool movement without fluoro***
- * ***advancing catheter without support***
- * ***advancing bgc without support***
- * ***tool vessel scraping***
- * ***nondistal tool vessel scraping***
- * ***lateral plane in use***
- * ***pushing after resistance increase***
- * ***pushing out wire when catheter toward vessel wall***

II-a Manipulation in the aortic arch using multipurpose catheter

Advance wire followed by the selective catheter until the ascending aorta at the level of the brachiocephalic trunk

- Advance 5F until visible

- Continue advancing wire followed by 5F until tip of catheter just proximal to brachiocephalic trunk

- * ***tool in side branch***
- * ***wire tip out of view***
- * ***distal tool movement without fluoro***
- * ***advancing catheter without support***
- * ***tool vessel scraping***
- * ***nondistal tool vessel scraping***
- * ***tool in aortic valve***
- * ***lateral plane in use***

III Access from arch to carotid

Manipulate slowly to locate and cannulate CCA

- Do a roadmap to identify target vessel, if neccessary

- Gently rotate and retract to probe for target vessel

Do a roadmap, with a large FOV, to identify target vessel, bifurcation and potential stenosis

- Make sure to have a good overview of the landing zone for the BGC

- If BGC still in aorta make sure to cover aortic arch

- If BGC already in CCA make sure to keep balloon in view

Navigate catheter over the wire into ICA (if no stenosis) until catheter in cervical carotid

Re-evaluate status by a full diagnostic run through catheter

- Use the right pedal to record a serie (a diagnostic run)

- Step on pedal, inject contrast and wait for venous filling before releasing pedal

* ***wire tip out of view***
* ***distal tool movement without fluoro***
* ***tool vessel scraping***
* ***nondistal tool vessel scraping***
* ***catheter too distal***
* ***wire too distal***
* ***forceful injection***
* ***advancing catheter without support***
* ***pushed retracting catheter***

IV Advancement and stable positioning of BGC

Advance the BGC over the catheter and position in a stable position in cervical ICA

- Advance BGC beyond carotid bulb

- Potition as distally as safely possible in cervical carotid

- Position balloon in a stable position in a straight segment

Remove wire and catheter (while maintaining position of BGC)

If you are satisfied with the position of the BGC, press 'place tool' to continue to next phase

- Click 'Place tool' button in the Inventory tab in the GUI

- Follow instructions on GUI screen and remove BGC completely out of the system

* ***wire tip out of view***
* ***bgc tip out of view***
* ***proximal tool movement without fluoro***
* ***distal tool movement without fluoro***
* ***pushing bgc while no tip progression***
* ***catheter retracting into cca***
* ***tool loosing access***
* ***forceful injection***
* ***forceful injection, filling contra M1 or BA***
* ***advancing catheter without support***
* ***balloon placed at carotid bulb***
* ***tool vessel scraping***
* ***nondistal tool vessel scraping***

V Tri-axial access to and crossing of thrombus

Shape the wire to facilitate navigating to and atraumatic crossing of thrombus

Insert DAC, MC and wire combination

First insert DAC until proximal to tip of BGC, then insert MC up to tip of DAC and last insert wire until protruding out of BGC

Select target vessel with wire and follow with MC and DAC

Ensure a working view suitable for microwire and MC navigation

- Magnify and center (FOV 10 in. or less), keeping BGC and occlusion in view

- Do a new roadmap

Stepwise advance wire and MC up to the thrombus, leaving DAC in distal ICA

Stepwise advance wire and MC to cross and pass the thrombus until M2-M3 transition preferably in a straight segment

Retrieve wire while maintaining position of MC

- ***bgc tip out of view***
- ***proximal tool movement without fluoro***
- ***distal tool movement without fluoro***
- ***pushing microcatheter while dac retracting***
- ***pushing microcatheter while buckling***
- ***bad roadmap***
- ***microwire forward movement out of catheter***
- ***forceful injection***
- ***forceful injection, filling contra M1 or BA***
- ***uncontrolled forward movement***
- ***no distal injection after perforation***
- ***advancing catheter without support***
- ***no distal injection after dissection***

VI Deployment of stentriever

Insert stentriever while maintaining position of MC until position recommended by IFU

Deploy according to IFU

- Advance retriever until distal tip aligns with tip of MC

- Retract MC until MC tip marker proximal to proximal marker of retriever

- Make sure to cover any bare wire of the stentriever

- After deploying retriever, visualize strut expansion and allow sufficient time for clot to integrate

- ***bgc tip out of view***
- ***proximal tool movement without fluoro***
- ***distal tool movement without fluoro***
- ***pushing microcatheter while buckling***
- ***stentriever forward movement during deployment***
- ***stentriever backward movement during deployment***
- ***stentriever deployed too distal***
- ***stentriever deployed too proximal***
- ***movement beyond initial mc tip***
- ***advancing catheter without support***
- ***movement of mc while inserting stentriever***
- ***uncontrolled forward movement***

VII Retrieval of thrombus

Achieve balloon occlusion

NOTE: Click the 'Inflation' button of the BGC in the Inventory tab to inflate the balloon

Get a more distal position of DAC by using stentriever as an anchor

- Apply a gentle backward tension on retriever while gently pushing DAC
- Make sure to not move or dislodge stentriever
- Stop when DAC at the level of the proximal marker of the stentriever
- Remove any forward tension in the system by retracting stentriever and DAC gently, as a unit

Start aspirating through DAC and BGC

NOTE: Aspiration is started by using the corresponding buttons in the Inventory tab

Retrieve stentriever into DAC under continued aspiration

- IF DAC covering proximal stetriever MC can be removed
- If DAC not reaching stentriever make sure MC covers any bare wire of the stentriever
- Use a smooth and continuous movement when retracting stentriever

Fully retrieve stentriever and clean under continued aspiration

- Use a smooth and continuous movement when retracting stentriever

If no flow through DAC, remove DAC under continued aspiration through DAC and BGC
If free flow through BGC, stop aspirating and deflate balloon

NOTE: Use the corresponding buttons in the Inventory tab

* ***bgc tip out of view***
* ***proximal tool movement without fluoro***
* ***distal tool movement without fluoro***
* ***balloon inflated at carotid bulb***
* ***balloon inflated in curve***
* ***balloon moving while inflated***
* ***balloon overinflated***
* ***balloon underinflated***
* ***stentriever moving without aspiration***
* ***Retrieving stentriever with an unprotected wire***
* ***advancing catheter without support***
* ***DAC passed m1***

VIII Angiographic control of success of retrieval

Do a non forceful injection through DAC if still inserted, otherwise through BGC, to control lesion
Verify reperfusion, if not successful reiterate from Phase V

- Follow insctructions on GUI screen
proximal tool movement without fluoro
distal tool movement without fluoro
forceful injection
advancing catheter without support
forceful injection, filling contra M1 or BA

IX Full head control angio

Full head angiogram (AP + Lateral, incl. full venous phase) through BGC
Check carotid unless included in post-thrombectomy angiogram
Retract BGC to proximal CCA and do a specific control angio through BGC

NOTE: Click 'Reactivate tool' in Inventory tab and follow instruction on fluoro screen
distal tool movement without fluoro
forceful injection
forceful injection, filling contra M1 or BA

X Safe removal of devices (and closure)

Remove all devices as a unit, leaving the BGC in abdominal aorta
Closure according to institutional protocol

- Finish exercise by Clicking 'Exit' in GUI

Table S2. EVT procedure Case 2 deconstructed into consecutive steps and possible handling errors.

** = Predefined possible handling errors*

I Femoral access to descending aorta

Advance wire in a smooth, continuous movement to mid thoracic aorta

- Advancing wire under fluoro, always keeping tip in view, until above celiac trunk and below left subclavian.

Advance BGC and selective catheter over the wire, under fluoroscopy, keeping tip of wire in view

- Advance BGC and 5F to mid thoracic aorta and proximal to flexible tip of wire

NOTE: In the simulator BGC must first be introduced to at least above celiac trunk before introducing the 5F

* ***tool in side branch***
* ***wire tip out of view***
* ***distal tool movement without fluoro***
* ***advancing catheter without support***
* ***advancing bgc without support***
* ***tool vessel scraping***
* ***nondistal tool vessel scraping***
* ***lateral plane in use***
* ***pushing after resistance increase***
* ***pushing out wire when catheter toward vessel wall***

II-b Manipulation in the aortic arch using a Simmon catheter

Advance wire and select the left subclavian artery

- Advance wire followed by 5F and select subclavian artery

- Advance wire beyond apex of subclavian artery

Advance the catheter over the wire into the subclavian artery until the sharp bend is well inside the artery

Retract wire proximal to the shoulder (the sharp bend of the catheter)

Form the reverse shape

- Make sure that the sharp bend of the catheter is retracted back to the aortic arch and is directed away from the subclavian bifurcation

- Rotate the catheter counter-clockwise and advance together with the wire as a unit

- If advancing into subclavian instead of aorta make sure bend is in aorta, adjust rotation slightly and retry.

- If not successful, consider slightly repositioning of the wire to get less or more support.

* ***wire tip out of view***
* ***distal tool movement without fluoro***
* ***tool vessel scraping***
* ***nondistal tool vessel scraping***
* ***lateral plane in use***
* ***advancing catheter without support***

III Access from arch to carotid

Manipulate slowly to locate and cannulate CCA

- Do a roadmap to identify target vessel, if neccessary
- Gently rotate and retract to probe for target vessel

Continue retracting and/or rotating as long as tip of catheter is advancing into target vessel (CCA)

Do a roadmap, with a large FOV, to identify target vessel, bifurcation and potential stenosis

- Make sure to have a good overview of the landing zone for the BGC
- If BGC still in aorta make sure to cover aortic arch
- If BGC already in CCA make sure to keep balloon in view

Navigate catheter over the wire into ICA (if no stenosis) until catheter in cervical carotid

Re-evaluate status by a full diagnostic run through catheter

- Use the right pedal to record a serie (a diagnostic run)
- Step on pedal, inject contrast and wait for venous filling before releasing pedal

- ***wire tip out of view***
- ***distal tool movement without fluoro***
- ***tool vessel scraping***
- ***nondistal tool vessel scraping***
- ***catheter too distal***
- ***wire too distal***
- ***forceful injection***
- ***advancing catheter without support***
- ***pushed retracting catheter***

IV Advancement and stable positioning of BGC

Advance the BGC over the catheter and position in a stable position in cervical ICA

- Advance BGC beyond carotid bulb
- Potition as distally as safely possible in cervical carotid
- Position balloon in a stable position in a straight segment

Remove wire and catheter (while maintaining position of BGC)

If you are satisfied with the position of the BGC, press 'place tool' to continue to next phase

- Click 'Place tool' button in the Inventory tab in the GUI
- Follow instructions on GUI screen and remove BGC completely out of the system

- ***wire tip out of view***
- ***bgc tip out of view***
- ***proximal tool movement without fluoro***
- ***distal tool movement without fluoro***
- ***pushing bgc while no tip progression***
- ***catheter retracting into cca***
- ***tool loosing access***
- ***forceful injection***
- ***forceful injection, filling contra M1 or BA***

* *advancing catheter without support*
* *balloon placed at carotid bulb*
* *tool vessel scraping*
* *nondistal tool vessel scraping*

V Tri-axial access to and crossing of thrombus

Shape the wire to facilitate navigating to and atraumatic crossing of thrombus

Insert DAC, MC and wire combination

First insert DAC until proximal to tip of BGC, then insert MC up to tip of DAC and last insert wire until protruding out of BGC

Select target vessel with wire and follow with MC and DAC

Ensure a working view suitable for microwire and MC navigation

- Magnify and center (FOV 10 in. or less), keeping BGC and occlusion in view
- Do a new roadmap

Stepwise advance wire and MC up to the thrombus, leaving DAC in distal ICA

Stepwise advance wire and MC to cross and pass the thrombus until M2-M3 transition preferably in a straight segment

Retrieve wire while maintaining position of MC

* *bgc tip out of view*
* *proximal tool movement without fluoro*
* *distal tool movement without fluoro*
* *pushing microcatheter while dac retracting*
* *pushing microcatheter while buckling*
* *bad roadmap*
* *microwire forward movement out of catheter*
* *forceful injection*
* *forceful injection, filling contra M1 or BA*
* *uncontrolled forward movement*
* *no distal injection after perforation*
* *advancing catheter without support*

no distal injection after dissection

VI Deployment of stentriever

Insert stentriever while maintaining position of MC until position recommended by IFU

Deploy according to IFU

- Advance retriever until distal tip aligns with tip of MC
- Retract MC until MC tip marker proximal to proximal marker of retriever
- Make sure to cover any bare wire of the stentriever
- After deploying retriever, visualize strut expansion and allow sufficient time for clot to integrate

* *bgc tip out of view*
* *proximal tool movement without fluoro*
* *distal tool movement without fluoro*
* *pushing microcatheter while buckling*
* *stentriever forward movement during deployment*

* ***stentriever backward movement during deployment***
* ***stentriever deployed too distal***
* ***stentriever deployed too proximal***
* ***movement beyond initial mc tip***
* ***advancing catheter without support***
* ***movement of mc while inserting stentriever***
* ***uncontrolled forward movement***

VII Retrieval of thrombus

Achieve balloon occlusion

NOTE: Click the 'Inflation' button of the BGC in the Inventory tab to inflate the balloon

Get a more distal position of DAC by using stentriever as an anchor

- Apply a gentle backward tension on retriever while gently pushing DAC
- Make sure to not move or dislodge stentriever
- Stop when DAC at the level of the proximal marker of the stentriever
- Remove any forward tension in the system by retracting stentriever and DAC gently, as a unit

Start aspirating through DAC and BGC

NOTE: Aspiration is started by using the corresponding buttons in the Inventory tab

Retrieve stentriever into DAC under continued aspiration

- IF DAC covering proximal stetriever MC can be removed
- If DAC not reaching stentriever make sure MC covers any bare wire of the stentriever
- Use a smooth and continuous movement when retracting stentriever

Fully retrieve stentriever and clean under continued aspiration

- Use a smooth and continuous movement when retracting stentriever

If no flow through DAC, remove DAC under continued aspiration through DAC and BGC
If free flow through BGC, stop aspirating and deflate balloon

NOTE: Use the corresponding buttons in the Inventory tab

* ***bgc tip out of view***
* ***proximal tool movement without fluoro***
* ***distal tool movement without fluoro***
* ***balloon inflated at carotid bulb***
* ***balloon inflated in curve***
* ***balloon moving while inflated***
* ***balloon overinflated***
* ***balloon underinflated***
* ***stentriever moving without aspiration***
* ***Retrieving stentriever with an unprotected wire***
* ***advancing catheter without support***
* ***DAC passed m1***

VIII Angiographic control of success of retrieval

Do a non forceful injection through DAC if still inserted, otherwise through BGC, to control lesion

Verify reperfusion, if not successful reiterate from Phase V

- Follow insctructions on GUI screen

* ***proximal tool movement without fluoro***
* ***distal tool movement without fluoro***
* ***forceful injection***
* ***advancing catheter without support***
* ***forceful injection, filling contra M1 or BA***

IX Full head control angio

Full head angiogram (AP + Lateral, incl. full venous phase) through BGC

Check carotid unless included in post-thrombectomy angiogram

Retract BGC to proximal CCA and do a specific control angio through BGC

NOTE: Click 'Reactivate tool' in Inventory tab and follow instruction on fluoro screen

* ***distal tool movement without fluoro***
* ***forceful injection***
* ***forceful injection, filling contra M1 or BA***

X Safe removal of devices (and closure)

Remove all devices as a unit, leaving the BGC in abdominal aorta

Closure according to institutional protocol

- Finish exercise by Clicking 'Exit' in GUI

Table S3. Comparison of the VR simulator parameters in pretest versus posttest for the two test cases, distributed between the centers SUS, SSK and AHUS.

		Case 1 (n = 19)			Case 2 (n = 17)		
		**SUS (n=10)**	**SSK (n=5)**	**AHUS (n=4)**	**SUS (n=8)**	**SSK (n=5)**	**AHUS (n=4)**
**Total time (min)**	**pretest**	39.4 (12.8)	29.3 (8.0)	24.0 (1.1)	31.5 (7.6)	26.4 (5.5)	28.9 (7.6)
	**posttest**	18.4 (3.3)	15.2 (2.6)	16.1 (1.9)	17.2 (4.4)	14.3 (2.0)	17.2 (1.9)
	**p-value**	< 0.001	0.014*	0.001	0.001*	0.002†	0.042
	**CI**	[-28.7, -13.3]	[-23.8, -4.3]	[-9.8, -6.0]	[-21.1, -7.4]	[-18.1, -6.0]	[-22.5, -0.8]
**Steps completed**	**pretest**	25.8 (7.5)	23.4 (5.1)	24.8 (7.0)	25.6 (9.6)	27.0 (6.4)	30.8 (4.9)
	**posttest**	32.6 (3.4)	29.0 (2.8)	31.3 (2.2)	38.4 (6.2)	32.2 (2.7)	36.5 (2.2)
	**p-value**	0.022*	0.01	0.16	0.008*	0.15*	0.12
	**CI**	[1.2, 12.4]	[2.3, 9.0]	[-4.6, 17.6]	[4.0, 21.4]	[-2.6, 13. 0]	[-2.2, 11.2]
**Handling errors**	**pretest**	25.9 (10.6)	61.8 (13.7)	15.3 (12.8)	23.4 (8.9)	52.8 (24.1)	36.5 (34.7)
	**posttest**	23.6 (13.1)	27.4 (8.6)	12.3 (2.8)	20.3 (19.0)	17.0 (13.5)	13.8 (17.0)
	**p-value**	0.67†	0.001	0.66†	0.67	0.002	0.092
	**CI**	[-13.5, 8.9]	[-44.6, -24.2]	[-19.1, 13.1]	[-19.9, 13.6]	[-50.3, -21.3]	[-52.4, 6.9]
**Contrast volume (ml)**	**pretest**	109.6 (39.6)	57.1 (34.3)	33.4 (17.4)	109.8 (53.1)	64.2 (25.6)	51.4 (27.2)
	**posttest**	75.1 (30.9)	30.4 (2.5)	46.0 (27.1)	63.5 (28.1)	33.2 (13.1)	48.7 (22.2)
	**p-value**	0.044†	0.16	0.47*	0.053*	0.028	0.88*
	**CI**	[-67.8, -1.1]	[-68.9, 15.6]	[-28.5, 53.7]	[-93.3, 0.6]	[-56.6, -5.4]	[-46.0, 40.6]
**Fluroscopy time (min)**	**pretest**	16.6 (8.0)	13.9 (7.0)	10.2 (0.9)	15.3 (0.9)	13.1 (3.3)	13.6 (6.3)
	**posttest**	7.4 (1.9)	6.7 (2.3)	6.6 (1.9)	7.6 (3.5)	5.7 (1.4)	8.5 (1.9)
	**p-value**	0.004	0.081*	0.029	0.007	0.004*	0.18
	**CI**	[-14.6, -3.6]	[-15.9, 1.3]	[-6.6, -0.7]	[-12.6, -2.9]	[-11.1, -3.4]	[-14.5, 4.1]
**Radiation dose (mGy)**	**pretest**	54.7 (40.9)	28.8 (21.2)	25.3 (4.6)	84.9 (66.9)	25.8 (10.4)	36.9 (16.4)
	**posttest**	25.7 (12.8)	11.3 (5.0)	19.8 (7.9)	20.6 (13.0)	8.9 (4.0)	17.7 (7.8)
	**p-value**	0.021	0.099	0.27	0.022	0.006	0.099*
	**CI**	[-52.3, -5.6]	[-40.0, 5.1]	[-18.5, 7.6]	[-116.2, -12.2]	[-25.7, -8.1]	[-44.5, 6.2]

All values are given in mean (standard deviation), n=numbers. CI = 95 % confidence interval. ml and mGy are fictive values calculated from the simulator. *= Welch t-test. † = Independent t-test

Table S4. Difference in variance between pretest and posttest of the VR simulator parameters for the two test cases.

	Case 1 (n = 19)			Case 2 (n = 17)		
	**pretest (SD)**	**posttest (SD)**	**p-value**	**pretest (SD)**	**posttest (SD)**	**p-value**
**Total time (min)**	33.5 (11.9)	17.1 (3.1)	<0.001	29.4 (7.0)	16.4 (3.5)	0.005
**Steps completed**	25.0 (6.5)	31.4 (3.3)	0.001	27.2 (7.7)	35.8 (5.2)	0.13
**Handling errors**	33.1 (21.3)	22.2 (11.6)	0.020	35.1 (23.9)	17.8 (16.3)	0.13
**Contrast volume (ml)**	79.7 (47.0)	57.2 (31.7)	0.060	82.7 (47.5)	51.1 (25.6)	0.13
**Fluroscopy time (min)**	14.6 (7.1)	7.0 (1.9)	0.001	14.3 (5.1)	7.2 (2.8)	0.015
**Radiation dose (mGy)**	41.7 (33.8)	20.7 (11.7)	0.011	56.2 (53.2)	16.5 (10.4)	0.005

The p-values in Table S4 refer to variance difference between pretest and posttest, while the p-values for the same data in Table 1 of the main article refers to mean difference between pretest and posttest.

All values are given in mean (standard deviation). P-values for difference in variance are calculated using Levene's test.

Table S5. Clinical results from 2019 to 2021, including all EVTs performed.

Center	Brain reperfusion (mTICI≥ 2b/3)	Independent at 3 months (mRS 0-2)	Hemorrhagic complications
SUS (n= 140)	84 %	39 %	4 %
SSK (n= 30)	83 %	43 %	3 %
AHUS (n= 41)	86 %	46 %	5 %

Including patients with pre-stroke mRS >2, end-stage cancer, or more than one EVT performed during the same admission.

n=number of patients. mTICI = Modified Treatment in Cerebral Ischemia Score. mRS = modified Rankin Scale score. sICH = Symptomatic Intracranial Hemorrhage.
